# Cathepsin W restrains peripheral regulatory T cells for mucosal immune quiescence

**DOI:** 10.1126/sciadv.adf3924

**Published:** 2023-07-12

**Authors:** Jian Li, Zuojia Chen, Girak Kim, Jialie Luo, Shohei Hori, Chuan Wu

**Affiliations:** ^1^ Experimental Immunology Branch, National Cancer Institute, NIH, Bethesda, MD 20892, USA.; ^2^ Laboratory of Immunology and Microbiology, Graduate School of Pharmaceutical Sciences, The University of Tokyo, Tokyo 113-0033, Japan.

## Abstract

Peripheral regulatory T (pT_reg_) cells are a key T cell lineage for mucosal immune tolerance and anti-inflammatory responses, and interleukin-2 receptor (IL-2R) signaling is critical for T_reg_ cell generation, expansion, and maintenance. The expression of IL-2R on pT_reg_ cells is tightly regulated to ensure proper induction and function of pT_reg_ cells without a clear molecular mechanism. We here demonstrate that Cathepsin W (CTSW), a cysteine proteinase highly induced in pT_reg_ cells under transforming growth factor–β stimulation is essential for the restraint of pT_reg_ cell differentiation in an intrinsic manner. Loss of CTSW results in elevated pT_reg_ cell generation, protecting the animals from intestinal inflammation. Mechanistically, CTSW inhibits IL-2R signaling in pT_reg_ cells by cytosolic interaction with and process of CD25, repressing signal transducer and activator of transcription 5 activation to restrain pT_reg_ cell generation and maintenance. Hence, our data indicate that CTSW acts as a gatekeeper to calibrate pT_reg_ cell differentiation and function for mucosal immune quiescence.

## Teaser

CTSW restrains pTreg cell differentiation and function for mucosal immune quiescence via inhibiting IL-2R signaling.

## INTRODUCTION

Naïve CD4^+^ T cells are capable of being polarized into different T cell subsets to fulfill their functions according to different tissue environment and inflammatory conditions. The full development and proper function of T cell subsets require their master transcription factors (TFs) to drive specific genetic programs. As one crucial machinery of tissue adaptation for CD4^+^ T cells, the induction, and maintenance of their identities are known to be tightly controlled for their optimal function (*1*, *2*). Different environmental cues, such as cytokine milieu, have been demonstrated to determine the commitment of specific T cell lineage (*3*). It has been acknowledged that, along with the expression of master TFs, other cell-intrinsic signaling cascades can also be induced or activated to secure T cell identity, preventing T cell fate deviation (*2*).

Regulatory T (T_reg_) cells represent a distinct group of CD4^+^ T cells with selective expression of TF forkhead box P3 (FOXP3) (*4*–*6*). They play a key role in controlling immune tolerance and quiescence (*7*, *8*). Based on their origins, two different subtypes of Foxp3^+^ T_reg_ cells have been identified: thymic T_reg_ (tT_reg_) cells and peripheral T_reg_ (pT_reg_) cells (*9*). tT_reg_ cells are known to develop in the thymus and recognize self-antigen, while pT_reg_ cells are differentiated from naïve CD4^+^ T cells in the peripheral organs and are essential for mucosal immune homeostasis on the barrier surfaces (*10*). Previous studies have shown that generation of both tT_reg_ and pT_reg_ cells requires T cell receptor (TCR) stimulation and cytokine signals, such as interleukin-2 (IL-2) (*11*, *12*). While TCR stimulation triggers nuclear factor κB signaling, IL-2 is known to activate signal transducer and activator of transcription 5 (STAT5) (*13*). Consequently, activation of the IL-2 signaling pathway contributes to the Foxp3 expression and T_reg_ cell development.

IL-2 binds with high affinity to IL-2 receptor (IL-2R), which is composed of IL-2R α chain (IL-2Rα; CD25), the common γ chain (γc; CD132), and IL-2Rβ (CD122) (*14*). Previous studies reveal the critical role of IL-2R for both tT_reg_ and pT_reg_ cell generation, maintenance, and expansion by stabilizing Foxp3 expression (*15*). Furthermore, IL-2R signaling has also been demonstrated to promote the suppressor function of T_reg_ cells via activation of STAT5 (*16*). As one most abundantly expressed subunit of IL-2R on T_reg_ cells, CD25 has been indicated to be coexpressed with Foxp3 in resting CD4^+^ T cells. Thus, CD25 can be used as a surface marker for T_reg_ cells, which is also recognized to be indispensable for T_reg_ cell development (*17*). Moreover, the expression of CD25 has been shown to contribute to both Foxp3 stability and suppressive capacity in T_reg_ cells (*18*). Loss of CD25 leads to impaired T_reg_ cell function, resulting in disrupted immune tolerance and autoimmunity (*19*–*21*). However, the expression of CD25 on T_reg_ cells displays a range of variety, depending on T_reg_ cell activation status and surrounding tissue environment. In addition, IL-2–IL-2R interaction transduces signal for the activation of STAT5, which determines Foxp3 expression (*22*). Although Foxp3 has been suggested to promote CD25 transcription (*23*), it is still largely unclear the molecular mechanism of how CD25 protein level is regulated for T_reg_ cell generation and development.

Cathepsin W (CTSW or lymphopain) is a member of the papain family of cysteine proteases (*24*). Most cathepsins are found in antigen-presenting cells and participate in antigen processing (*25*). Previous studies show that CTSW can be found in CD8^+^ T cells and natural killer (NK) cells, while its role in controlling immune responses has not been clearly elucidated (*26*). In the current study, through transcriptomic and proteomic profiling, we identified that CTSW was highly induced in pT_reg_ cells in the presence of transforming growth factor–β (TGF-β), which was essential for the restraint of pT_reg_ cell differentiation and function in a cell-intrinsic manner. Loss of CTSW leads to elevated Foxp3 expression during pT_reg_ cell differentiation with enhanced suppressive function. CTSW deficiency also resulted in increased pT_reg_ cells on the mucosal surface of the gastrointestinal (GI) tract in vivo. Mechanistically, we demonstrated that CTSW participated in processing cytosolic IL-2R by interacting with and cleaving CD25. Consequently, CTSW limited STAT5 activation under IL-2 signaling, restraining Foxp3 expression and pT_reg_ cell function. Therefore, we here illustrate negative machinery to confine pT_reg_ cell generation via restraint of IL-2R signaling by CTSW, calibrating the mucosal immune balance for intestinal physiology.

## RESULTS

### CTSW is highly expressed by Smad3 in pT_reg_ cells under TGF-β stimulation

**Fig. 1.**
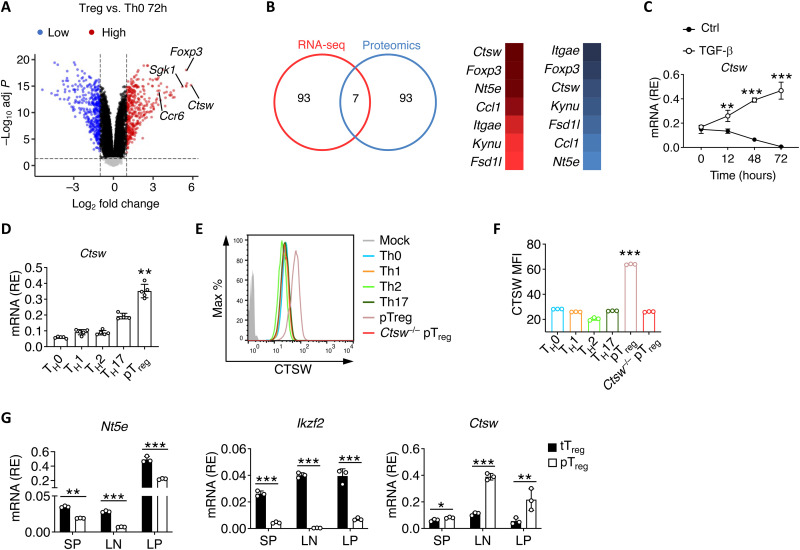
Cathepsin W (CTSW) is highly expressed in peripheral regulatory T (pT_reg_) cells under transforming growth factor–β (TGF-β) stimulation. (**A**) Volcano plot comparing the *P* value versus fold change for reads of mRNA sequencing (mRNA-seq) data from 72-hour in vitro–differentiated pT_reg_ cells versus T_H_0 cells. The key genes of T_reg_ cells are highlighted in red (transcripts up-regulated in T_reg_ cells). (**B**) Top 100 genes of mRNA-seq analysis of TGF-β–induced genes in CD4^+^ T cells stimulated with TGF-β for 48 hours overlap with top 100 proteins from proteomic analysis on in vitro–differentiated pT_reg_ cells (ProteomeXchange Consortium identifier: PXD007826) (left). Fold change ranking top seven genes in mRNA-seq (red) and proteomic analysis (blue) (right). (**C**) Temporal expression of *Ctsw* in naïve CD4^+^ T cells under TGF-β stimulation. RE, relative expression value; Ctrl, control. (**D**) Quantitative polymerase chain reaction (qPCR) analysis of CTSW expression in different T cell subsets during in vitro differentiation. (**E** and **F**) (E) Representative flow cytometry analysis and (F) quantification of CTSW expression in different T cell subsets during in vitro differentiation. MFI, mean fluorescence intensity. (**G**) qPCR analysis of the tT_reg_ cell signature genes *Nt5e*, *Ikzf2*, and *Ctsw* in tT_reg_ cells (CD4^+^Foxp3^+^Nrp1^+^) and pT_reg_ cells (CD4^+^Foxp3^+^Nrp1^−^) isolated from the spleen (SP), peripheral lymph nodes (LNs), and lamina properia (LP) of wild-type (WT) mice. Data are representative of three independent experiments (A to G). **P < 0.01 and ***P < 0.001 [(C) two-way analysis of variance (ANOVA) with Tukey’s multiple comparisons test; (D and F) one-way ANOVA with Tukey’s multiple comparisons test; (G) Student’s *t* test; error bars represent SD].

To identify the potential regulators that are critical for pT_reg_ cell generation, we differentiated naïve CD4^+^ T cells with or without TGF-β stimulation for 72 hours and subjected them to mRNA sequence. We noticed highly expressed *Ctsw* in pT_reg_ cells (Fig. 1A). Furthermore, overlapping top 100 most up-regulated genes between our 72-hour pT_reg_ mRNA sequencing data and published proteomics data from in vitro–differentiated pT_reg_ cells (*27*) also identified *Ctsw* as a potential target (Fig. 1B). These results suggest that CTSW could be induced during pT_reg_ cell development. We then verified the inducible expression of *Ctsw* under TGF-β stimulation by kinetic quantitative polymerase chain reaction (qPCR) assessment (Fig. 1C). CTSW was found to be higher in pT_reg_ cells than that in other T cell subsets during in vitro differentiation (Fig. 1, D to F). To determine whether CTSW was also highly expressed in pT_reg_ cell in vivo, we analyzed the expression of CTSW in Foxp3^+^ T_reg_ cells from wild-type (WT) mice positive or negative for neuropilin-1 (Nrp1) (*28*, *29*). Consistent with the in vitro results, pT_reg_ cells (CD4^+^Foxp3^+^Nrp1^−^) isolated in vivo exhibited a higher expression of *Ctsw* than that of tT_reg_ cells (CD4^+^Foxp3^+^Nrp1^+^) isolated from various compartments (Fig. 1G). Together, these data demonstrate that CTSW was expressed specifically in pT_reg_ cells both in vitro and in vivo.

**Fig. 2.**
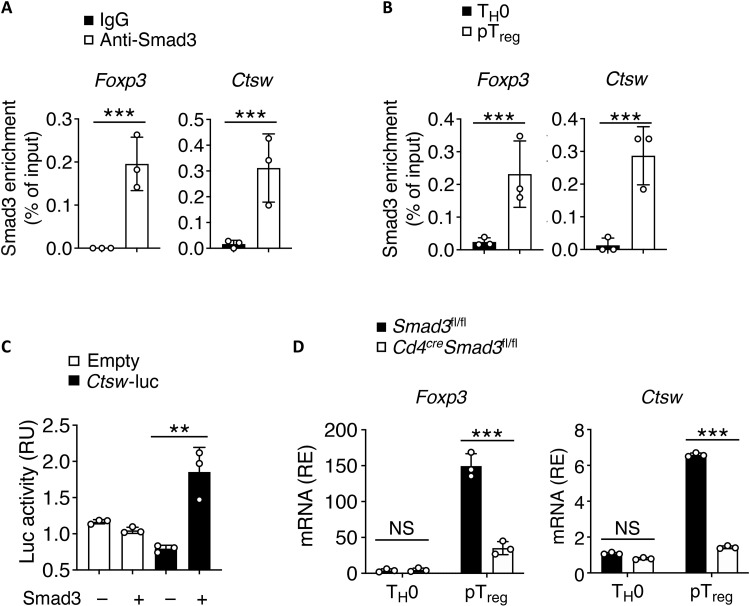
Smad3 drives *Ctsw* expression during peripheral regulatory T (pT_reg_) cell differentiation. (**A** and **B**) Chromatin immunoprecipitation followed by PCR (ChIP-PCR) analysis of the abundance of Smad3 bound to the *Foxp3* and *Ctsw* promoter (A) with anti-Smad3 or control antibodies and (B) in T_H_0 and pT_reg_ cells. IgG, immunoglobulin G. (**C**) Luciferase activity in Jurkat T cells transfected with an empty luciferase reporter (empty) or a *Ctsw* promoter–driven luciferase reporter (*Ctsw*-luc) together with a Smad3 or control vector (−). RU, relative luciferase unit. (**D**) qPCR analysis of *Foxp3* and *Ctsw* mRNA in WT or Smad3 deficient pT_reg_ cells. Data are representative of three independent experiments (A to D). NS, not significant; **P < 0.01 and ***P < 0.001 [(C) one-way ANOVA with Tukey’s multiple comparisons test; (A, B, and D) Student’s *t* test; error bars represent SD].

TGF-β–mediated pT_reg_ cell differentiation and Foxp3 expression depend on Smad3 signaling pathway (*30*, *31*). We examined whether Smad3 can directly regulate CTSW expression under TGF-β stimulation. Chromatin immunoprecipitation followed by PCR (ChIP-PCR) analysis showed that, within differentiated pT_reg_ cells, Smad3 was found to interact with *Ctsw* promoter (Fig. 2A). Furthermore, such binding was not observed under T helper 0 (T_H_0) conditions (Fig. 2B). By luciferase assay, we confirmed that Smad3 transactivated *Ctsw* (Fig. 2C). Conditional deletion of Smad3 in CD4^+^ T cells led to more reduced *Foxp3* and *Ctsw* in pT_reg_ cells than that of their counterparts from control cells (Fig. 2D). These data indicate that CTSW expression was directly promoted by Smad3 under TGF-β signaling.

### CTSW intrinsically restrains pT_reg_ cell differentiation

**Fig. 3.**
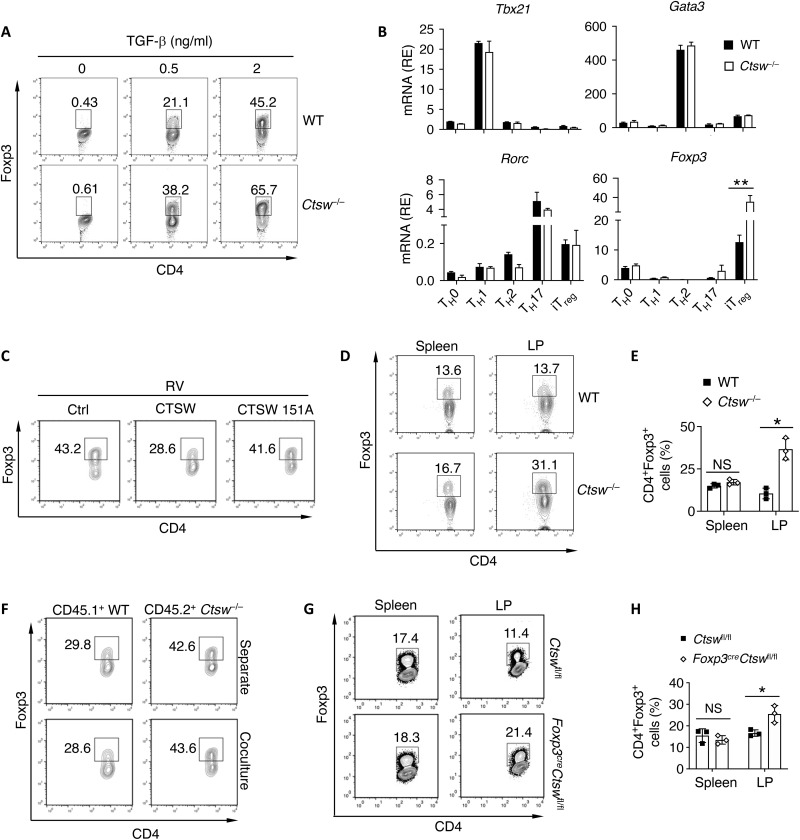
Cathepsin W (CTSW) intrinsically restrains peripheral regulatory T (pT_reg_) cell differentiation. (**A**) Representative flow cytometry analysis of Foxp3 expression in naïve WT and *Ctsw*^−/−^ CD4^+^ T cells under stimulation of different dosages of transforming growth factor–β (TGF-β) for 72 hours. (**B**) Quantitative polymerase chain reaction (qPCR) analysis of indicated genes in different T cell subsets during in vitro differentiation. (**C**) Naïve CD4^+^ T cells retroviral transduced with WT CTSW and proteolytic inactive (CTSW 151A) form in the presence of TGF-β. Representative flow cytometry analysis of Foxp3 expression. (**D** and **E**) (D) Representative flow cytometry analysis and (E) quantification of Foxp3 expression in CD4^+^ cells in the spleen and lamina properia (LP) of WT and *Ctsw*^−/−^ mice. (**F**) CD45.1^+^ WT and CD45.2^+^ *Ctsw*^−/−^ naïve T cells were cultured separately or together in the presence of TGF-β. The frequency of Foxp3^+^ cells was then determined by flow cytometry. (**G** and **H**) (G) Representative flow cytometry analysis and (H) quantification of Foxp3 expression in CD4^+^ cells in the spleen and LP of *Foxp3^cre^Ctsw*^fl/fl^ and control mice. Data are representative of two independent experiments (A to H). *P < 0.05 and **P < 0.01 (Student’s *t* test; error bars represent SEM).

We next examined the role of CTSW in pT_reg_ generation. We found CTSW deficiency led to elevated Foxp3 expression under TGF-β stimulation (Fig. 3A). Meanwhile, loss of CTSW did not induce defects of differentiation within other T cell subsets (fig. S1A). qPCR analysis verified that CTSW deficiency resulted in specific up-regulation of Foxp3 in pT_reg_ cell but no altered expression of master TFs in other T cell subsets (Fig. 3B). Moreover, retroviral overexpression of CTSW repressed Foxp3 expression in the presence of TGF-β during in vitro differentiation while such effect was abolished by a proteolytic inactive mutant of CTSW (CTSW C151A) (Fig. 3C), suggesting that proteolytic activity of CTSW is crucial for its function in regulating pT_reg_ cells. Next, we investigated the role of CTSW for pT_reg_ cell development in vivo. We observed that CTSW-deficient mice displayed normal T cell development, including CD4^+^ and CD8^+^ T cell proportion and naïve CD4^+^ cell frequency in the peripheral lymphoid organs (fig. S1, B to E). It has been described that pT_reg_ cells are mainly generated and accumulate in the GI tract (*10*). Although we found a similar frequency of Foxp3^+^ T_reg_ cells in the spleen, *Ctsw*^−/−^ mice exhibited enhanced T_reg_ cells in the gut compared to WT controls (Fig. 3, D and E). Furthermore, we found an increased RORγt^+^Helios^−^ pT_reg_ population in the GI compartment in *Ctsw*^−/−^ compared to WT mice (fig. S1, F and G). Together, these results suggest that CTSW inhibits pT_reg_ cell generation both in vitro and in vivo.

We next asked whether CTSW regulates pT_reg_ cell differentiation via an intrinsic or extrinsic mechanism. We polarized CD45.1^+^ WT and CD45.2^+^ *Ctsw*^−/−^ naïve CD4^+^ T cells in the presence of TGF-β either separately or together. The difference of Foxp3 expression between WT and *Ctsw*^−/−^ CD4^+^ T cells was retained when the two types of cells were cultured together, suggesting that CTSW regulates T_reg_ cell differentiation in an intrinsic manner (Fig. 3F). Furthermore, we generated *Ctsw*^fl/fl^ (fig. S2A) mice and crossed them with *Foxp3^cre^* strain (*32*) to deplete CTSW specifically in T_reg_ cells (fig. S2B). Naïve CD4^+^ T cells from *Foxp3^cre^Ctsw*^fl/fl^ mice also exhibited elevated pT_reg_ cell differentiation in vitro compared to those from control mice (fig. S2C). We found increased T_reg_ cell in the GI compartment but not in the spleen in *Foxp3^cre^Ctsw*^fl/fl^ mice compared to control mice (Fig. 3, G and H, and fig. S2, D and E), indicating that environmental CTSW has little impact on T_reg_ cell generation in vivo.

### CTSW deficiency promotes intestinal pT_reg_ generation

**Fig. 4.**
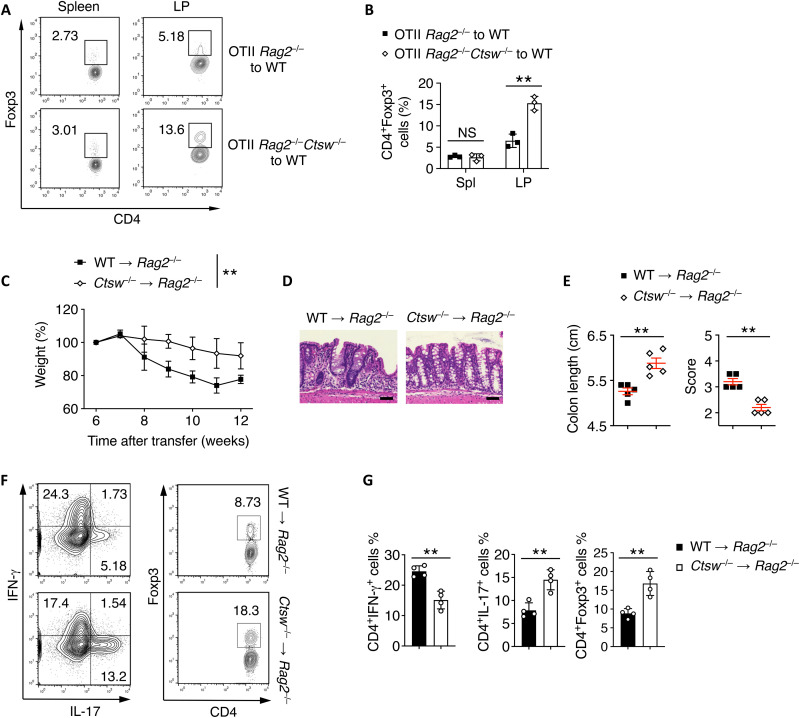
Cathepsin W (CTSW) deficiency promotes intestinal peripheral regulatory T (pT_reg_) generation. (**A** and **B**) OTII *Rag2*^−/−^ and OTII *Rag2*^−/–^*Ctsw*^−/−^ CD45.2^+^CD4^+^ T cells were transferred into congenic CD45.1 WT recipient mice, followed by administration of ovalbumin (OVA) in the drinking water for 5 days. (A) Representative flow cytometry analysis and (B) quantification of CD45.2^+^Foxp3^+^ pT_reg_ cells from the spleen (Spl) and lamina properia (LP) in the recipient mice. (**C** to **G**) CD4^+^CD45RB^hi^CD25^−^ T cells were intraperitoneally transferred into *Rag2*^−/−^ mice for colitis induction. Mice were euthanized and analyzed 10 weeks after colitis induction. (C) Body weight of *Rag2*^−/−^ mice received WT or *Ctsw*^−/−^ T cells during colitis. (D) Hematoxylin and eosin staining of colon samples from the different groups as in (C). Scale bars, 50 μm. (E) Colon lengths (left) and histological score (right) from the different groups as in (C). (F to G) (F) Representative flow cytometry and (G) quantification of IL-17 and interferon-γ (IFN-γ) production, Foxp3 expression by CD4^+^ T cells isolated from LP from indicated groups as in (C). Data are representative of three independent experiments (A to G). **P < 0.01 [(B, E, and G) Student’s *t* test; (C) two-way ANOVA with Tukey’s multiple comparisons test; error bars represent SD].

pT_reg_ cells are critical for mucosal immune homeostasis, particularly in the GI tract (*10*). To further investigate the effect of CTSW in controlling pT_reg_ cell generation in vivo, we carried out a model of ovalbumin (OVA)–induced oral tolerance. We purified OVA-specific naïve CD4^+^ T cells from OTII *Rag2*^−/−^ mice with or without deletion of CTSW and transferred them into congenic CD45.1 WT recipient mice. In response to OVA from drinking water as an oral antigen, the frequency of Foxp3^+^ T_reg_ cells in the CD45.2^+^ donor cells was increased specifically from lamina properia (LP) in the absence of CTSW compared to those from WT donors (Fig. 4, A and B). We next examined the role of CTSW for T_reg_ cell generation during intestinal inflammation. Either WT or *Ctsw*^−/−^ CD4^+^CD25^−^CD45RB^hi^ cells were transferred into *Rag2*^−/−^ mice to induce colitis. Mice that received *Ctsw*^−/−^ CD4^+^CD25^−^CD45RB^hi^ cells exhibited protected disease with less colonic tissue damage compared to WT donor cells (Fig. 4, C to E). Recovered *Ctsw*^−/−^ T cells from colonic tissues showed increased Foxp3^+^ T_reg_ cells than that of WT T cells (Fig. 4, F and G). We also found reduced interferon-γ (IFN-γ) expression levels from *Ctsw*^−/−^ T cells compared to WT T cells (Fig. 4, F and G), suggesting that loss of CTSW promotes T_reg_ generation and suppressed inflammatory responses.

### CTSW-deficient pT_reg_ cells exhibit enhanced suppressive activity

**Fig. 5.**
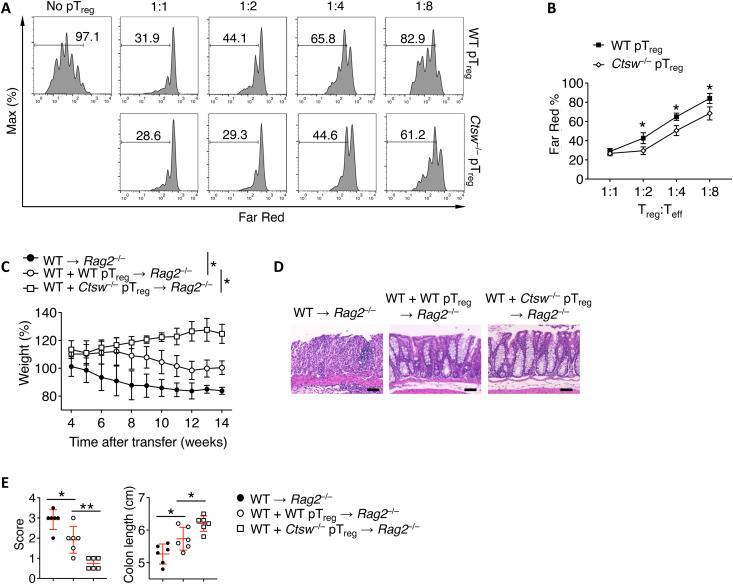
Cathepsin W (CTSW)–deficient peripheral regulatory T (pT_reg_) cells exhibit enhanced suppressive activity. (**A** and **B**) Proliferation of CellTrace Far Red–labeled WT naïve CD4^+^ T cells in the presence of anti-CD3, irradiated CD4^+^ T cell–depleted splenocytes, and green fluorescent protein (GFP)^+^ (Foxp3^+^) pT_reg_ cells from *Foxp3*^GFP^ and *Foxp3*^GFP^*Ctsw*^−/−^ mice. Data shown are determined by (A) flow cytometry and (B) quantification. T_eff_, effector T cells. (**C**) Body weight of *Rag2*^−/−^ mice transferred with WT CD4^+^CD45RB^hi^CD25^−^ T cells with or without WT or *Ctsw*^−/−^ Foxp3^+^ pT_reg_ cells. (**D**) Hematoxylin and eosin staining of colon samples from the different groups as in (C). Scale bars, 50 μm. (**E**) Colon lengths (left) and histological score (right) from the different groups as in (C). Data are representative of three independent experiments (A to D) or are pooled from two independent experiments (E). *P < 0.05 [(B and C) two-way ANOVA with Tukey’s multiple comparisons test; (E) one-way ANOVA with Tukey’s multiple comparisons test; error bars represent SD].

Next, we asked whether there is a functional alteration of pT_reg_ cells in the absence of CTSW. We crossed CTSW-deficient mice with *Foxp3*^GFP^ reporter mice to obtain naïve CD4^+^ T cells and exclude green fluorescent protein (GFP)^+^ tT_reg_ cells from the analysis. Naïve CD4^+^ T cells (CD4^+^CD62L^+^CD44^−^GFP^−^) from the WT and *Ctsw*^−/−^ mice were differentiated in vitro into pT_reg_ cells for 3 days, and GFP^+^ pT_reg_ cells were sorted and then assessed in functional assays. We observed an increased level of suppressive capacity in *Ctsw*^−/−^ pT_reg_ cells compared to that of WT T_reg_ cells (Fig. 5, A and B). The enhanced regulatory function of *Ctsw*^−/−^ pT_reg_ cells was further evaluated in a T cell cotransfer colitis model. WT CD4^+^CD25^−^CD45RB^hi^ cells were transferred into *Rag2*^−/−^ mice, together with either WT or *Ctsw*^−/−^ in vitro–differentiated Foxp3^+^ (GFP) pT_reg_ cells at a ratio of 10:1. We observed that *Ctsw*^−/−^ pT_reg_ cells exhibited better capacity in suppressing the development of intestinal inflammation than WT pT_reg_ cells as measured by weight loss (Fig. 5C) and tissue pathology (Fig. 5, D and E). Collectively, these data suggest that CTSW deficiency results in enhanced pT_reg_ cell suppressive function in protecting mucosal tissue inflammation.

### CTSW deficiency enhances pT_reg_ cell stability

**Fig. 6.**
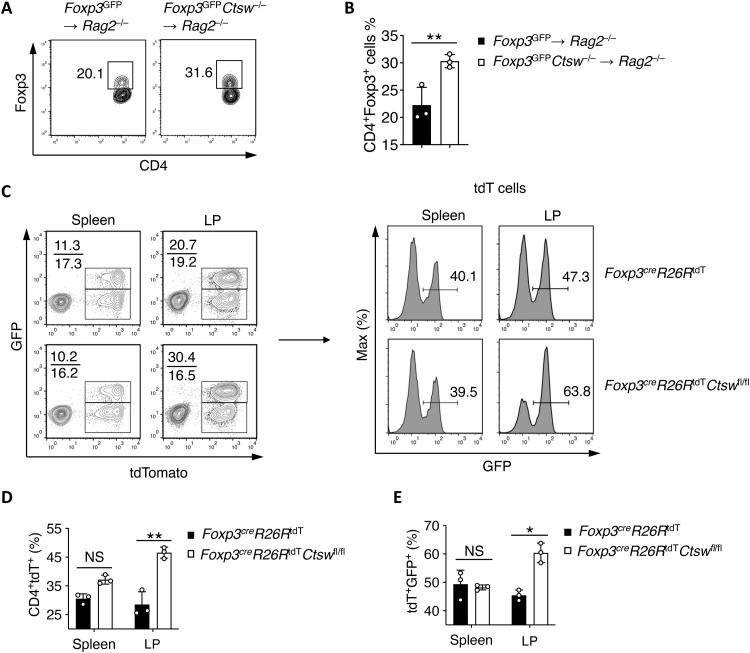
Cathepsin W (CTSW) deficiency enhances peripheral regulatory T (pT_reg_) cell stability. (**A** and **B**) Purified green fluorescent protein (GFP)^+^ pT_reg_ cells from *Foxp3*^GFP^ and *Foxp3*^GFP^*Ctsw*^−/−^ mice were intravenously transferred into *Rag2*^−/−^ recipient mice. Five days after transfer, the frequency of GFP^+^ cells was determined by (A) flow cytometry and (B) quantification. (**C**) GFP expression within CD4^+^tdTomato^+^ T cells isolated from the spleen and LP of *Foxp3^cre^R26R*^tdT^ fate-mapping (FM) mice with or without CTSW deficiency was determined by flow cytometry. (**D** to **E**) Quantification of the frequency of (D) CD4^+^tdTomato^+^ and (E) tdTomato^+^GFP^+^ T cells in the spleen and lamina properia (LP) of WT and CTSW-deficient FM mice. Data are representative of three independent experiments (A to E). *P < 0.05 and **P < 0.01 (Student’s *t* test; error bars represent SD).

Given the feature of T_reg_ cell plasticity (*33*), we further evaluated whether CTSW affected pT_reg_ cell stability. We transferred purified in vitro–differentiated GFP^+^ WT or *Ctsw*^−/−^ pT_reg_ cells into *Rag2*^−/−^ mice. By analyzing the presence of Foxp3^+^ (GFP) cells 5 days after transfer, we found that there was a higher frequency of Foxp3^+^ cells in recipients of *Ctsw*^−/−^ donor cells (Fig. 6, A and B), implicating that the loss of CTSW elevated pT_reg_ cell stability in vivo. Next, we used a Foxp3 fate-mapping (FM) mouse by crossing the *Foxp3^cre^* (Foxp3-GFP-cre) mice (*32*) with *Rosa26*^tdT^ (Rosa26 genetically targeted of loxP-stop-loxP-tdTomato) mice. This allows us to trace the cells that have transcribed Foxp3 expression regardless of their present production of Foxp3. We observed enhanced both CD4^+^ tdTomato^+^ and CD4^+^ tdTomato^+^GFP^+^ cells in the gut but not in the spleen with the CTSW-deficient FM mice (Fig. 6, C to E), suggesting increased stability of pT_reg_ cells in maintaining Foxp3 expression in the intestine in the absence of CTSW.

### Loss of CTSW leads to altered IL-2–STAT5 signaling pathway in pT_reg_ cells

**Fig. 7.**
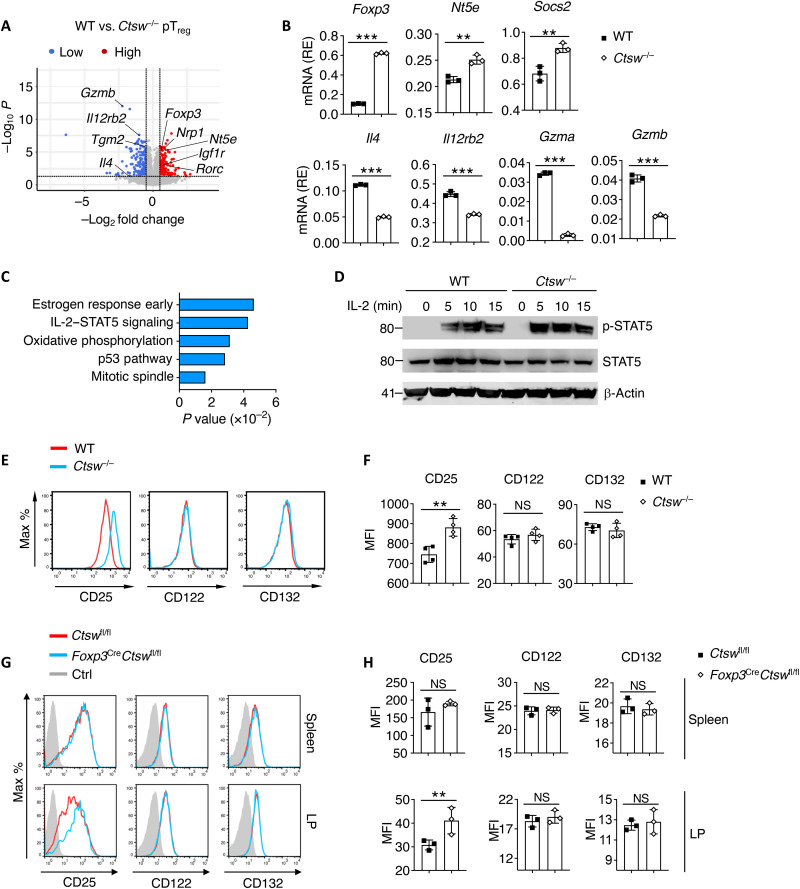
Loss of cathepsin W (CTSW) leads to compromised IL-2–STAT5 signaling pathway in peripheral regulatory T (pT_reg_) cells. (**A**) Volcano plot comparing the *P* value versus fold change for reads of mRNA sequencing (mRNA-seq) data from in vitro–differentiated T_reg_ cells from WT versus *Ctsw*^−/−^ mice. The genes of significant change are highlighted in red (transcripts up-regulated in *Ctsw*^−/−^ pT_reg_ cells) and in blue (transcripts down-regulated in *Ctsw*^−/−^ pT_reg_ cells), respectively. (**B**) Quantitative polymerase chain reaction (qPCR) analysis of indicated genes from WT and *Ctsw*^−/−^ pT_reg_ cells. (**C**) Gene set enrichment analysis of up-regulated signaling pathway in *Ctsw*^−/−^ pT_reg_ cells. (**D**) Immunoblot analysis of p-STAT5, STAT5, and β-actin in WT and *Ctsw*^−/−^ in vitro–differentiated pT_reg_ cells with IL-2 stimulation. (**E** and **F**) (E) Representative flow cytometry analysis and (F) quantification of CD25, CD122, and CD132 expression on WT and *Ctsw*^−/−^ in vitro–differentiated Foxp3^+^ pT_reg_ cells. (**G** and **H**) (G) Representative flow cytometry analysis and (H) quantification of CD25, CD122, and CD132 on Foxp3^+^ T_reg_ cells from the spleen and LP of *Foxp3^cre^Ctsw*^fl/fl^ and control mice. Data are representative of two independent experiments (A to H). **P < 0.01 and ***P < 0.001 [(B, F, and H) Student’s *t* test; error bars represent SD].

To investigate the molecular mechanisms of how CTSW regulates pT_reg_ cell generation and function, we performed mRNA sequencing and found that a set of genes regulated by Foxp3 was differentially expressed in CTSW-deficient compared to control pT_reg_ cells (Fig. 7A). Expression of selected genes was further confirmed by qPCR (Fig. 7B). Furthermore, signaling pathway analysis revealed that IL-2–STAT5 pathway was highly up-regulated in CTSW-deficient pT_reg_ cells (Fig. 7C and fig. S3A). We found a hyperactivation of STAT5 in CTSW-deficient pT_reg_ cells under IL-2 stimulation compared to control pT_reg_ cells (Fig. 7D). Furthermore, we noticed up-regulated IL-2 targeted genes in CTSW-deficient than WT T_reg_ cells (fig. S3B). IL-2R plays an essential role in T_reg_ cell development and function via IL-2–STAT5 signaling pathway (*21*). In addition, both IL-2R complex and CTSW could be found in the subcellular compartment of endoplasmic reticulum (ER) (*34*, *35*). Thus, we hypothesized that the proteolytic activity of CTSW might affect IL-2R levels, regulating Foxp3 expression and pT_reg_ cell differentiation. By examining different IL-2R subunits, we found elevated CD25 but not CD122 and CD132 expression on CTSW-deficient pT_reg_ cells in vitro (Fig. 7, E and F). We also found that Foxp3^+^ T_reg_ cells exhibited enhanced CD25 in the colon but not in the spleen of *Foxp3^cre^Ctsw*^fl/fl^ mice compared to the control mice (Fig. 7, G and H). Thus, our data suggest that CTSW is critical for CD25 expression and IL-2–STAT5 signaling during pT_reg_ cell generation.

### Cytosolic CTSW-CD25 interaction restrains pT_reg_ cell generation

**Fig. 8.**
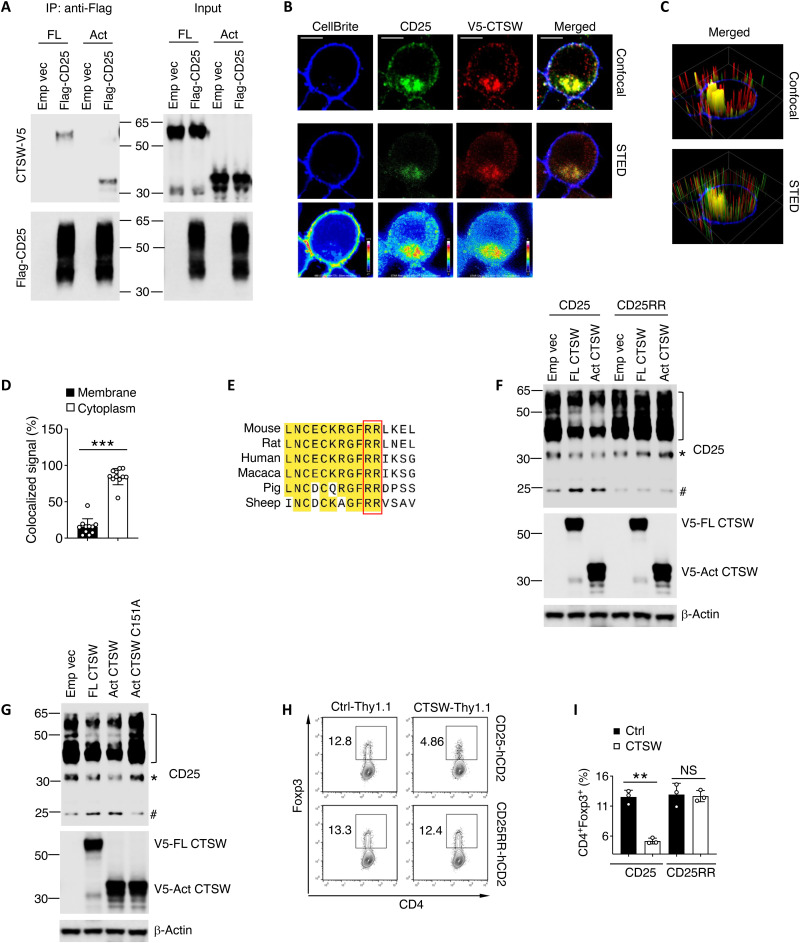
Cytosolic cathepsin W (CTSW)–CD25 interaction determines peripheral regulatory T (pT_reg_) cell generation. (**A**) Co-immunoprecipitation (IP) of CTSW and CD25 from extracts of cotransfected 293 T cells. HEK293 T cells were transfected with mouse V5-tagged active form and inactive form of CTSW and Flag-tagged CD25. Anti-V5 immunoprecipitates (IP) and total lysates were immunoblotted with anti-V5 and anti-Flag antibodies. (**B**) Naïve CD4^+^ T cells retroviral transduced with V5-tagged CTSW in the presence of transforming growth factor–β (TGF-β). Confocal and stimulated emission depletion (STED) imaging of CD25 and V5 on in vitro–differentiated pT_reg_ cells were shown. Heat imaging was generated on the basis of STED microscopy (bottom row). (**C**) CD25 and V5 signals in (C) were reconstructed to three-dimensional illustration. (**D**) Quantification of colocalization CD25 and V5 on the cell membrane and cytoplasm as exemplified in (C). (**E**) Amino acid alignment illustrating conservation in CTSW cleave motif across different species. (**F**) HEK293 T cells were cotransfected with mouse V5-tagged CTSW and Flag-tagged CD25. Total lysates were immunoblotted with anti-CD25 and anti-V5 antibodies (], glycosylated CD25; *, nonglycosylated CD25; #, cleaved CD25). (**G**) HEK293 T cells were cotransfected with indicated forms of V5-tagged CTSW and Flag-tagged CD25. Total lysates were immunoblotted with anti-CD25 and anti-V5 antibodies (], glycosylated CD25; *, nonglycosylated CD25; #, cleaved CD25). (**H** and **I**) Naïve CD25-deficient CD4^+^ T cells retroviral transduced with CTSW and CD25 or CD25RR in the presence of TGF-β. (H) Representative flow cytometry analysis and (I) quantification of Foxp3 expression. Data are representative of two independent experiments (A to C and E to I) or are pooled from two independent experiments (D). **P < 0.01 and ***P < 0.001 [(D) Student’s *t* test; (I) two-way ANOVA with Tukey’s multiple comparisons test; error bars represent SD].

To gain insight of how CTSW processes CD25 for pT_reg_ cell generation, we performed co-immunoprecipitation (IP) and found that CTSW and CD25 formed a protein complex (Fig. 8A). By using stimulated emission depletion (STED) microscopy, we found that most CTSW-CD25 interactions were localized within the cytoplasm instead of the cell surface of pT_reg_ cells (Fig. 8, B to D). To further analyze the proteolysis of CD25 by CTSW, we predicted the potential cutting site based on human CTSW cutting site preference in MEROPS protease database (*36*). We noticed that one site on the extracellular domain of mouse CD25 matched one of the CTSW cutting site preference patterns near the N terminus, which is conserved across different species (Fig. 8E). We found that mutation of the cleavage site in CD25 (substitute both CD25 R56 and R57 to alanine, CD25RR) protected CD25 protein from proteolytic cleavage, suggesting the important role CTSW for the protein integrity of CD25 (Fig. 8F). To determine whether a decreased CD25 expression level is due to CTSW proteolytic activity, we expressed CD25 along with different forms of CTSW in human embryonic kidney (HEK) 293 T cells. Compared to active form of CTSW, inactive form of CTSW C151A abolished its function in cleaving CD25 (Fig. 8G). These results indicated that cytosolic CTSW binds to and processes CD25. We next investigated whether CTSW restricts pT_reg_ cell differentiation through cutting CD25 by using a CTSW-resistant form of CD25. To this end, retrovirally overexpressed CD25RR in CD25-deficient T cells diminished the effect of CTSW in repressing Foxp3 expression during pT_reg_ cell differentiation compared to WT CD25 overexpression (Fig. 8, H and I). These data demonstrate that CTSW restrains pT_reg_ cell differentiation through proteolytic processing CD25.

## DISCUSSION

pT_reg_ cells are known to be enriched at the barrier surfaces in the presence of TGF-β (*10*, *37*). Various extrinsic and intrinsic factors have been illustrated to control pT_reg_ cell generation and function (*13*). Our previous study also demonstrated that it is critical to the silence of unspecific transcriptional programs for other T cell subsets to ensure the unidirectional development of pT_reg_ cells for mucosal immune homeostasis (*38*). In the current study, we highlighted a proteinase CTSW, specifically induced during pT_reg_ cell development, that functions as a negative regulator in restraining pT_reg_ cell generation via cleaving IL-2R subunit CD25. We further illustrated that CTSW dampens the function and stability of pT_reg_ cells such that loss of CTSW in T_reg_ cells promotes the resistance of inflammatory responses.

CTSW is previously found to be highly expressed in CD8^+^ T cells and NK cells (*26*). Our data here show specific induction of CTSW in pT_reg_ cells that can be induced by TGF-β stimulation. We further elucidate that Smad3 directly promotes *Ctsw* transcript. Although the roles of CTSW in CD8^+^ T and NK cells are still under investigation, induction of CTSW in CD8^+^ T and NK cells could also be mediated via TGF-β–Smad3 signaling given that TGF-β and Smad3 have been reported to be crucial for effector function in both cell types (*39*, *40*). Furthermore, enhanced expression of CTSW was observed in cytotoxic cells during autoimmune gastritis, while two predominant pro-inflammatory cytokines, tumor necrosis factor–α and IFN-γ, have little effect in regulating CTSW (*41*, *42*). Considering that elevated TGF-β was noticed in both gastric cancer and intestinal inflammation (*43*), it could function as an inducer for CTSW in CD8^+^ T cells. In addition, IL-2 is known to promote CTSW expression (*34*), implicating that IL-2R-CTSW could form a negative feedback loop for pT_reg_ cell homeostasis given that Foxp3 promotes CD25 expression (*23*).

Transfer of CTSW-deficient T cells into *Rag2*^−/−^ mice led to enhanced levels of pT_reg_ cells, associated with elevated IL-17–producing cells. The role of T_H_17 and IL-17A in the pathogenesis of colitis is still controversial (*44*, *45*). Intestinal T_H_17 abundance is not only regulated by T_reg_ cells, but also gut microenvironment, such as microbiota. Abundance of pT_reg_ cells in the gut can also result in increased T_H_17 cells (*46*). Moreover, it has been reported that, instead of their direct effect, T_H_17-derived T_H_1 cells are required for the pathogenesis of colitis (*47*). In addition, intestinal T_reg_ cells suppress the conversion of T_H_17 cells to colitogenic T_H_1 cells, resulting in the accumulation of T_H_17 cells (*48*). Thus, it is possible that CTSW deficiency–enhanced pT_reg_ cell differentiation and function lead to elevated T_H_17 cells in the gut during inflammation.

Subcellular CTSW is found to locate on ER (*34*), which potentially agrees with our data of its intrinsic role in restraining pT_reg_ cell generation. While it has been suggested that intracellular CTSW can be packed into secretory granules and secreted upon activation (*26*), our data provide evidence that CTSW might exert its function via an intrinsic manner. It has also been demonstrated that IL-2R starts to assemble in the ER. IL-2–IL-2R signaling cascade could take place even when receptors are not on cell membranes (*35*). Because CD122 and CD132, but not CD25, can be quickly degraded after IL-2R engagement with IL-2 and internalization (*49*, *50*), a negative mechanism might be also needed to control CD25 protein level.

It has been well documented that IL-2 signaling plays an essential role in the development and function of T_reg_ cells. Disruption of different components in IL-2 signaling pathway, including CD25, CD122, Janus kinase 3, and STAT5, leads to compromised T_reg_ generation associated with autoimmune diseases (*21*, *51*). In addition, overexpression of a constitutively active form of STAT5b (STAT5b-CA) not only can enhance T_reg_ cell generation but also can rescue the impaired Foxp3 expression due to the loss of IL-2R (*21*, *52*). Lined up with these findings, our data suggest that CTSW-mediated IL-2R cleavage reduces STAT5 activation for the development of T_reg_ cells. Given that IL-2R-STAT5 signaling has been shown to be indispensable for the suppressive function and maintenance of pT_reg_ cells (*15*, *21*, *52*–*54*), our current findings consistently show that CTSW-mediated CD25 cleavage affects both pT_reg_ cell stability and suppressive capacity.

The differentiation and function of pT_reg_ cells on the barrier surfaces are precisely calibrated, which is crucial for the maintenance of mucosal immune homeostasis. CTSW functions as a key negative regulator induced under TGF-β stimulation to restrain the pT_reg_ cell generation via cleavage of CD25. Our current study highlights that CTSW intrinsically suppresses pT_reg_ cells, suggesting that repressing CTSW activity by a small-molecule inhibitor could be a therapeutic approach to enhance pT_reg_ cell expansion, initiating potent anti-inflammatory responses for tissue protection.

## MATERIALS AND METHODS

### Study design

This study aimed to figure out the potential regulators that are critical for pT_reg_ cell generation. Ctsw was identified through transcriptomics and proteomics profiling. The expression level of Ctsw mRNA and protein was confirmed in different T cell subsets by qPCR and flow cytometry, in in vitro T cell differentiation, and in vivo pT_reg_ induction model. The transcriptional regulation of Ctsw was analyzed by ChIP-PCR and luciferase assay. We generated the *Ctsw^flox/flox^* mice and characterized the T cell compartments in the mice with Ctsw-specific deletion in T_reg_ cells. The function of T_reg_ cells was investigated in in vitro suppressive assay and in vivo T cell transfer colitis model. The stability of T_reg_ cells was also evaluated in T_reg_ cell transfer experiments and FM reporter mice. Moreover, we performed mRNA sequencing and examined the IL-2/Stat5 signaling pathway in T_reg_ cells by flow cytometry. The interaction between Ctsw and CD25 was further confirmed by protein IP and immunofluorescence assays in retroviral infected pT_reg_ cells. The number of mice and statistics used in the studies are included in the figure legends.

### Mice

C57BL/6J (WT), *Cd4*^*cre*^, B6.SJL-*Ptprc^a^Pepc^b^*/J (CD45.1), *Foxp3*^GFP^, *Rosa26*^tdT^, *IL-2R*α^−/−^, *Rag2*^−/−^, and *OTII* mice were purchased from the Jackson Laboratory. *Foxp3^cre^* mice were described previously (*32*). *Smad3^flox^* mice were obtained from S. G. Rane (National Institute of Diabetes and Digestive and Kidney Diseases) (*55*). *Ctsw*^−/−^ mice were obtained from C. Pham (Washington University School of Medicine) (*56*).

The *Ctsw^flox^* mice were generated using the CRISPR-Cas9 method targeting the exon 2 of the Ctsw genome (*57*). The guide RNAs (gRNAs) and donor DNA were designed by National Cancer Institute (NCI) Genome Modification Core. Briefly, multiple gRNAs targeting 5′ and 3′ of exon 2 were designed using single-guide RNA (sgRNA) Scorer 2.0 (*58*) and tested for cutting activity as previously described (*59*). Two chemically modified sgRNAs (sgRNA-1: CAAGGTACAGGCGTCATCCTGGG; sgRNA-2: TGTACTTATGCTTTCGGATGGGG) were obtained from Synthego. The single-stranded donor DNA containing loxp (gtctccctaggaacagatttgtctttataccccagaatagtttggagcctgacagccaagagctgctttcgaaaagaaatgggtggggttagccaggcaggcaggtggagggtctcccctcctccctaagcctggtttccccctcgtggtttctccagccccgccctttccagcttagtctacacagcttcctgcctctagcttagcacttgtggctgcaccatgacactgactgcccacctctcctactttctggtcctgttgttagcgggccaaggcctcagtgactccctcctcaccaaggtaaatgtttctaacctgctttccatacccactccacctctggggtcttttctttatgaactggcatatttttcttcttcctttttaattttcttaagacagggtcccgaagggcttggtagcagaaggatctgtgagttcgagaccagcctggtctatgtagtgaggacagggctatgaggagagaccttaagacaaaggtctccctttgtagctcaggttagcttttaactgtgtagcccagaatgcttcactggcttggaactctcagtcgtcctcctgcctcagcctcttgaatgctggaatgagcattcaactggaggggtgagctgtcctgtctagctttaccgtcccgacttctcagagggaactgtgtggatataacttcgtatagcatacattatacgaagttatgctcacatttagaacagagaacaaaacaggatgacgcctgtaccttggagataagtttggggtcacaataatttttcccccacttttggttccttgccaggatgcaggtccccggccactggagctgaaggaagtcttcaagctgttccagatccggttcaaccggagttactggaacccagcaggtaccacgggcacagatgttcccagtctgacttctgctgtccacttaagagatgtacttatgctttcggatggttatagacctgggccaggccactgagtataacttcgtatagcatacattatacgaagttatgggaactaggatctgtagtgtactccttagcttccccgatgcatgatgggaatggaatgatccccagggtctatagccacttaaccatgactttaggcagggtgcggaggactgccatattgtcacatattcaatcagtagatccgtcttcaaggtggcaaccccagtcccaagctaccataaagggcaggcttgctctctctcccccaccttctcttttcttctgtctcctctctctccctccccacttctgtgtctgtgcgccaaaccccacctacccatcctccaaacctgtggtcagatgtgtgaagaggagacagagatggtgcagcccacgggtgcaggaaactggcctccagccactgttaactttcctttctgagttactgcctctaaaggctttgctcggcttccccctagagtgggcctgttagtgtctctggggaaaattctaatctcatgtggggctggataatcactttcttgcaggagagttgtagtgacagaaagtgccagcacttacctgagtgggtgtgggtggcccaaccagtggccacttcctgccctgagactggcctacagcaactcttttttggtctagagtacactcgccgtctgagcatctttg) was generated using a Guide It Long ssDNA production kit. The two gRNAs and donor DNA were co-microinjected with Cas9 protein into the cytoplasm of fertilized eggs collected from C57BL/6N mice by the NCI Transgenic Mouse Model Laboratory. Offspring born to the foster mothers were genotyped by PCR and Sanger sequencing. Founder mice with the desired insertion were bred with C57BL/6 mice to establish the mouse line.

Mouse lines were interbred in our facility to obtain the final strains. Mice were maintained at the NCI facility under specific pathogen–free conditions. Mice were fed a standard chow diet and used at 7 to 12 weeks of age for most experiments. All experiments were carried out in accordance with the guidelines prescribed by the Institutional Animal Care and Use Committee at the NCI.

### In vitro T cell differentiation

Naïve (CD44^lo^CD62L^+^CD25^−^) CD4^+^ T cells were obtained from the spleens and lymph nodes of indicated mice by fluorescence-activated single-cell sorting (BD FACSAria). The purity of isolated T cell populations routinely exceeded 98%. Naïve T cells were stimulated with plate-bound anti-CD3 (145-2C11, 1 μg/ml) and anti-CD28 (PV-1, 1 μg/ml) and polarizing cytokines (R&D Systems): TGF-β (2 ng/ml) for pT_reg_, IL-12 (20 ng/ml) for T_H_1, IL-4 (20 ng/ml) for T_H_2, TGF-β (2 ng/ml), and IL-6 (20 ng/ml) for T_H_17. Cells were cultured for 72 hours before analysis. For pT_reg_ cell stimulation by IL-2, the differentiated pT_reg_ cells were transferred into another plate for rest overnight, and IL-2 (0.1 ng/ml) was added for an indicated time.

### pT_reg_ cell generation in vivo

CD45.1^+^ mice were transferred with 2.5 × 10^6^ naïve T cells from OTII *Rag2*^−/−^ and *Ctsw^−/−^*OTII *Rag2*^−/−^ mice by intravenous injection. After 24 hours, the mice were given drinking water containing 3% OVA (Sigma-Aldrich) for 5 days.

### Flow cytometry

For intracellular cytokine staining, cells were polarized as described above and stimulated for 4 hours at 37°C in a culture medium containing phorbol 12-myristate 13-acetate (50 ng/ml; Sigma-Aldrich), ionomycin (1 μg/ml; Sigma-Aldrich), and monensin (GolgiStop; 1 μg/ml; BD Biosciences). Surface molecules were stained in phosphate-buffered saline (PBS) with 1% fetal calf serum for 20 min at room temperature, then fixed and permeabilized with the Intracellular Fixation & Permeabilization Buffer Set according to the manufacturer’s instructions (Invitrogen), and stained with cytokine antibodies diluted in Perm/Wash. For Foxp3, RORγt, and Heilos stating, cells were fixed and permeabilized with the Foxp3 Staining Buffer Set, according to the manufacturer’s instructions (Invitrogen). All flow cytometry data were acquired on the BD LSRFortessa X-20 Analyzer and analyzed with FlowJo software. Additional information on antibodies is listed in Table S1.

### Suppression assay

Naïve CD4^+^ T effectors (1 × 10^5^) were cultured with pT_regs_ (titrated down as described) in 96-well round-bottom plates (Corning). T effector cells were labeled with CellTrace Far Red (Invitrogen) and stimulated with anti-CD3 and irradiated CD4 cell–depleted splenocytes for 72 hours. CellTrace Far Red signals were detected by flow cytometry.

### T cell–induced colitis

CD4^+^CD25^−^CD45RB^hi^ cells were purified by fluorescence-activated cell sorting from WT or *Ctsw*^−/−^ mice and injected intraperitoneally at 7 × 10^5^ with or without in vitro–differentiated and resorted GFP^+^ WT or *Ctsw*^−/−^ pT_reg_ CD4^+^ Foxp3^+^ (7 × 10^4^) into age- and sex-matched *Rag2*^−/−^ mice, and weight loss was monitored.

### Histology

Mouse intestine tissues were fixed in 10% formalin and preserved in 70% ethanol. Samples were then paraffin-embedded and cut into 10-μm longitudinal sections, and the hematoxylin and eosin staining was performed by Histoserv Inc. (Germantown, MD). Pathology was scored from 0 to 5 in a blinded fashion: score 0: no changes observed; score 1: minimal scattered mucosal inflammatory cell infiltrates, with or without minimal epithelial hyperplasia; score 2: mild scattered to diffuse inflammatory cell infiltrates, sometimes extending into the submucosa and associated with erosions, with minimal to mild epithelial hyperplasia and minimal to mild mucin depletion from goblet cells; score 3: mild to moderate inflammatory cell infiltrates that were sometimes transmural, often associated with ulceration, with moderate epithelial hyperplasia and mucin depletion; score 4: marked inflammatory cell infiltrates that were often transmural and associated with ulceration, with marked epithelial hyperplasia and mucin depletion; score 5: marked transmural inflammation with severe ulceration and loss of intestinal glands.

### Quantitative reverse transcription PCR

RNA was extracted with RNAeasy mini-kits (Qiagen), and RNA was reverse-transcribed to complementary DNA (cDNA) using the iScript cDNA Synthesis Kit (Bio-Rad) according to the manufacturer’s instructions. The cDNA samples were used at 20 ng per well in a 384-well plate and run in triplicate. PCR reactions were set up using the SYBR Universal PCR Master Mix (Applied Biosystems) on the ABI Prism 7500 Sequence Detection System. Quantification of relative mRNA expression was normalized to the expression of β*-Actin*, and the relative expression value was shown as the result. The primers used are listed in Table S2.

### Expression plasmid construction and transfection

The mammalian expression vectors pCMV-Flag and pCMV-V5, retroviral expression vectors Murine Stem Cell Virus (MSCV)–internal ribosomal entry site (IRES)–Thy1.1 and MSCV-IRES-hCD2, and packaging plasmids pCL and Gag/pol were kept in our laboratory. The Ctsw and its mutations were amplified from the Ctsw expression plasmid from C. Pham (*56*). The CD25 and its mutation were amplified from mouse CD25 cDNA clone (Sino Biological). All the PCR reactions were performed with the Phusion High-Fidelity PCR Master Mix (New England Biolabs), and the sequence was verified by Sanger sequencing (Quintara Biosciences). The plasmids were transfected into HEK293 T cells with Lipofectamine 3000 (Invitrogen), and cells were harvested for analysis 48 hours after transfection. The primers used for PCR amplification are listed in Table S3.

### Western blot analysis

Cells (2 × 10^6^ to 5 × 10^6^) were lysed in a radioimmunoprecipitation assay (RIPA) buffer [20 mM tris-HCl (pH 8.0), 150 mM NaCl, 0.25% sodium deoxycholate, 1 mM EDTA, 1% NP-40, and protease inhibitors]. Proteins were separated by SDS–polyacrylamide gel electrophoresis gel electrophoresis using 4 to 12% NuPAGE bis-tris gels (Invitrogen) followed by transfer to nitrocellulose membrane. Membranes were incubated with 5% milk in Tris-buffered saline with 0.1% Tween® 20 detergent (TBST) [0.5 M NaCl, tris-HCl (pH 7.5), and 0.1% Tween 20] for 60 min to block unspecific binding. Proteins of interest were detected by incubating membranes overnight at 4°C in a blocking buffer with anti-Flag (Sigma-Aldrich), anti-V5 (Abcam), or anti–β-actin (Sigma-Aldrich), washing three times with TBST for 10 min and incubating with horseradish peroxidase–conjugated anti-rabbit or anti-mouse antibody (Cell Signaling Technology). Blotting signaling was detected with the SuperSignal West Pico PLUS Chemiluminescent Substrate (Thermo Fisher Scientific).

### Immunoprecipitation

Cell lysates were prepared as described above. Dynabeads Protein G (Invitrogen) was preincubated with 2 μg of antibody according to the manufacturer’s instructions. Proteins were immunoprecipitated by incubation of lysates with beads overnight at 4°C, beads were washed extensively with RIPA buffer, and proteins were boiled with a NuPAGE lithium dodecyl-sulfate (LDS) sample buffer (10% β-mercaptoethanol). The presence of immunocomplexed proteins was determined by Western blot analysis with the antibodies indicated.

### Chromatin immunoprecipitation

Experiments were performed using the SimpleChIP Enzymatic Chromatin IP Kit according to the manufacturer’s instructions. Antibody anti-Smad3 (5 μg; Abcam, ab28379) was used to precipitate Smad3-DNA complex in each ChIP assay. The regions that were identified by ChIP-PCR were amplified by the following primers used for qPCR: *Ctsw-30 to Ctsw-170*: CCAGCTTAGTCTACACAGCTTCC and CCAGTTCATAAAGAAAAGACCCCA; *Foxp3 CNS1:* CAGAGGTCAAAAGTGTGGGTATG and ACTTGAGTTGAGGCTAGGTTGTTC.

### Retroviral transduction

Retroviral particles were produced by transiently transfecting HEK293 T cells with retroviral packaging (pCL and gag-pol) and plasmids MSCV-IRES-GFP, MSCV-CTSW-IRES-GFP, or MSCV-CD25-IRES-hCD2 using Lipofectamine 3000 (Invitrogen). Seventy-two hours after transfection, viral culture supernatants were harvested, supplemented with polybrene (8 mg/ml; MilliporeSigma), and used to transduce previously stimulated T cells (5 × 10^5^ per well, plate-bound anti-CD3 and anti-CD28 and TGFβ for 24 hours). Cultures were centrifuged at 800*g* for 45 min at 25°C to facilitate infection. Cells were further cultured for 48 hours before analysis.

### Ctsw promoter luciferase reporter assay

A fragment of the *Ctsw* promoter [−2000 to +500 base pairs (bp) relative to the transcription start site] was amplified by PCR from genome DNA purified from splenocytes with the primers CATTTCTCTGGCCTAACTGGCCGGTACCCAAGATCCAAAGAGGACT and TCTTGATATCCTCGAGGCTAGCGTTCTCTGTTCTAAATGTGAGCA. Obtained DNA sequence was inserted between Kpn I and Nhe I in pGL3-Basic (Promega). Jurkat cells (4 × 10^6^ cells per cuvette) were electroporated on a BTX-600 machine using a Biorad 0.4-cm cuvette at 260 V, 1050 μF, and 720 ohms. Smad3-expressing plasmid (10 μg) or empty vector control along with the promoter firefly luciferase-reporter constructs and Renilla luciferase reporter vector (Promega) were transfected in each cuvette. Thirty hours after transfection, luciferase expression was determined by measuring luminescence with the Dual-Luciferase Reporter Assay System (Promega). The firefly luciferase activity was normalized to renilla luciferase activity, and the relative luciferase unit was shown as the result. Data are representative of at least two independent experiments; each data point represents duplicate values.

### Immunofluorescence

Purified naïve CD4^+^ T cells from WT were stimulated with anti-CD3 and anti-CD28 antibodies (1 μg/ml) in the presence of TGF-β for 24 hours and then transduced with CTSW-expressing retrovirus. Forty-eight hours later, cells were stained with CellBrite to stain cell membrane for 12 min at 37°C. Cells were then resuspended and spun on polylysine-treated glass slide and followed by fixation and permeabilizing. After blocking with 5% bovine serum albumin in PBS–Triton X-100 (0.1% in PBS), cells were incubated with anti-CD25 and anti-V5 at 4°C overnight. On the next day, slides were washed and incubated with anti-rat Abberior STAR Orange and anti-mouse Abberior STAR Red. Last, slides were mounted using Fluoromount-G (Invitrogen). Cells were observed on an inverted Nikon Ti-S microscope plus STEDYCON with NIS-Eleents imaging software (Nikon Instruments). The image was analyzed by Fiji ImageJ.

### RNA-seq and data analysis

The purified RNA was used for preparing RNA sequencing (RNA-seq) libraries with the Illumina TruSeq RNA Sample Preparation Kit. Libraries were sequenced with Illumina Nextseq 500 75-bp pair-end (PE) by Sequencing Facility at NCI. Raw data were analyzed with limma, Gimma, and egdeR packages in RStudio (version 1.4.1106) using R (version 4.0.4) to obtain differentially expressed genes. Gene set enrichment analysis was performed using Clusterprofiler package and mouse_H_v5 database.

### Statistical analysis

Data were analyzed using GraphPad Prism 9.0 (GraphPad software, La Jolla, CA, USA). Depending on the experimental design, statistical significance was tested via two-tailed unpaired Student’s *t* test. *P* values of <0.05 were considered significant (*P < 0.05, **P < 0.01, and ***P < 0.001); *P* > 0.05, nonsignificant. Details on the test used can be found in the respective figure legends.

## Notes

View/request a protocol for this paper from *Bio-protocol*.
