# Supplementary material for: Cathepsin W restrains peripheral regulatory T cells for mucosal immune quiescence
Source: Sci Adv. 2023 Jul 12;9(28):eadf3924. doi: 10.1126/sciadv.adf3924 (PMC10337914; doi:10.1126/sciadv.adf3924)
Supplement: Supplementary file 1 — Figs. S1 to S3 Tables S1 to S3 [file sciadv.adf3924_sm.pdf]

Science Advances
AAAS

Supplementary Materials for

Cathepsin W restrains peripheral regulatory T cells for mucosal immune quiescence

Jian Li *et al.*

Corresponding author: Chuan Wu, chuan.wu@nih.gov

Sci. Adv. **9**, eadf3924 (2023)
DOI: 10.1126/sciadv.adf3924

This PDF file includes:

Figs. S1 to S3
Tables S1 to S3

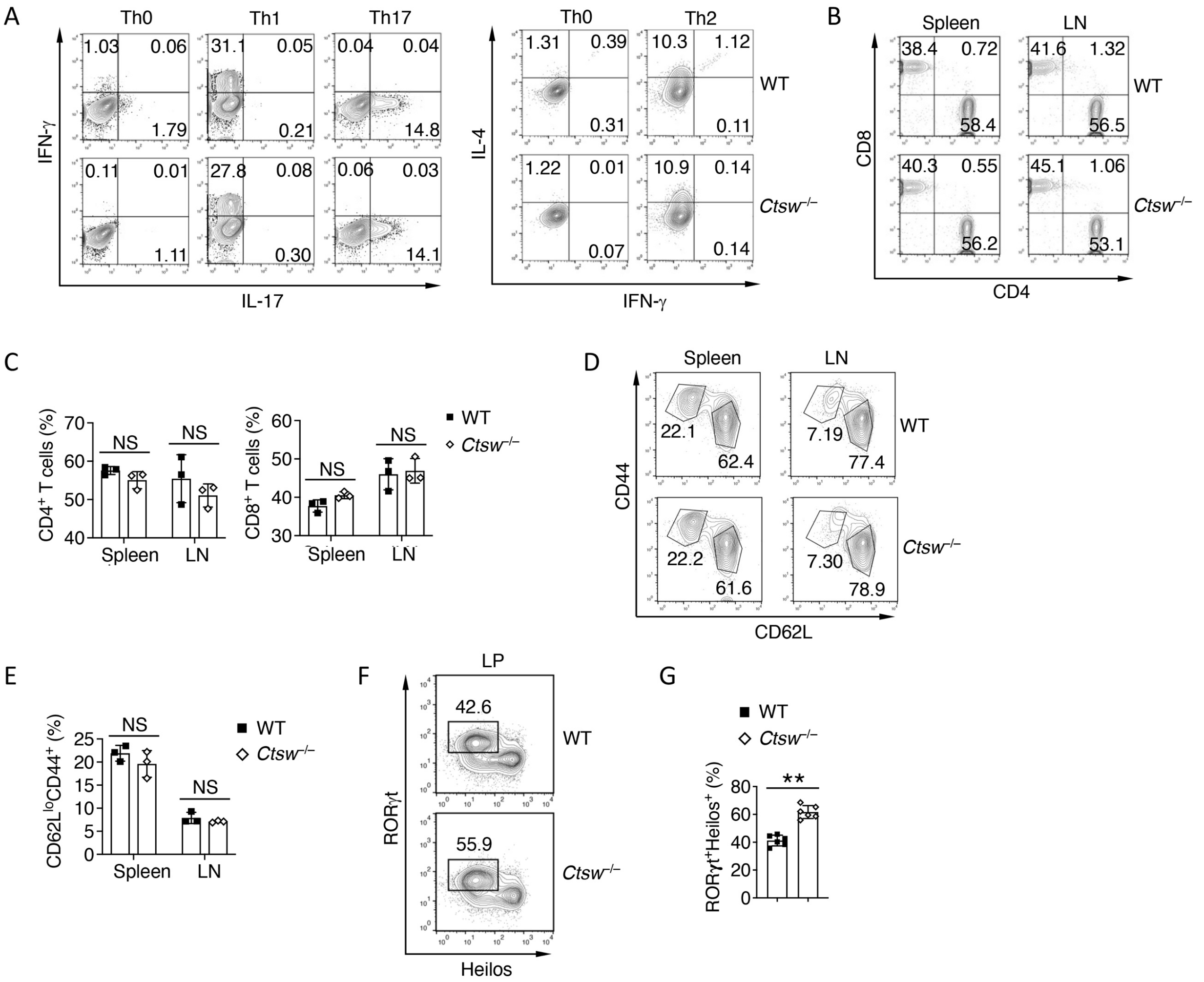

Fig S1. CTSW deficiency does not impact T cell development.

(A) Naive $CD4^+$ T cells from WT and *Ctsw*$^{-/-}$ mice were stimulated under Th0, Th1, Th2, Th17 conditions and harvested at 72 hours. Intracellular staining of indicated cytokines produced by different polarized T cell subsets cells from WT and *Ctsw*$^{-/-}$ mice was determined by flow cytometry.

(B-C) (B) Representative flow cytometry and (C) quantification of CD4 and CD8 expression in $CD45^+$ lymphocytes from spleen and peripheral lymph nodes (LN) of 8 weeks old WT and *Ctsw*$^{-/-}$ mice.

(D-E) (D) Representative flow cytometry and (E) quantification of CD62L and CD44 expression in $CD4^+$ T cells from spleen and LN of 8 weeks old WT and *Ctsw*$^{-/-}$ mice.

(F-G) (F) Representative flow cytometry analysis and (G) quantification of RORγt and Heilos expression in $CD4^+Foxp3^+$ cells in LP of WT and *Ctsw*$^{-/-}$ mice.

Data are representative of at least two independent experiments (A-G). NS, not significant, **p<0.01, (Student's *t*-test, error bars represent SD).

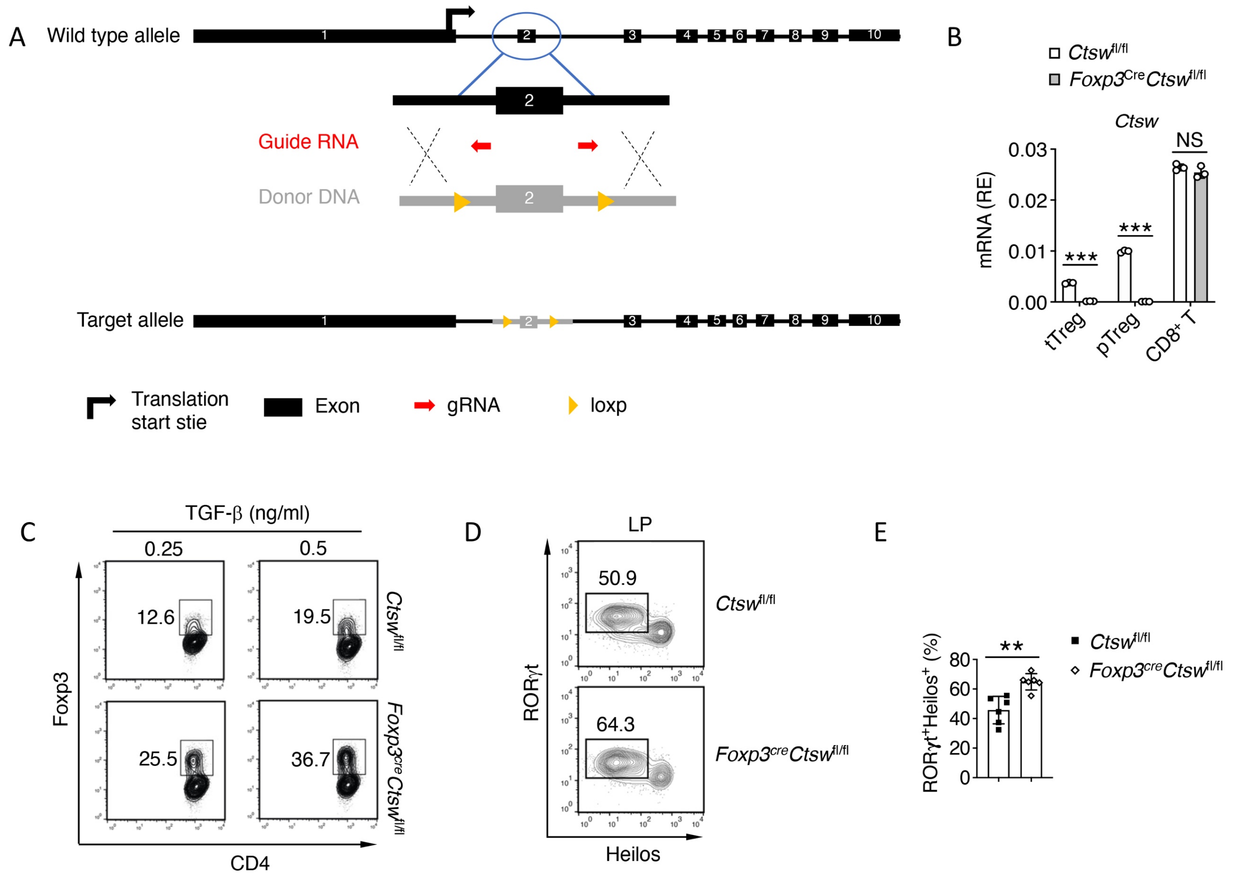

Fig S2. CTSW intrinsically restrains pTreg cell differentiation.

(A) Schematic illustration of *Ctsw*$^{fl/fl}$ mice generation.

(B) tTreg cells (CD4$^+$Foxp3$^+$Nrp1$^+$), pTreg cells (CD4$^+$Foxp3$^+$Nrp1$^-$) and CD8+ T cells were isolated from the lymph nodes of *Foxp3*^{cre}*Ctsw*$^{fl/fl}$ and control mice. qPCR analysis of *Ctsw* were performed in different T cell subsets.

(C) Representative flow cytometry analysis of Foxp3 expression in naive *Foxp3*^{cre}*Ctsw*$^{fl/fl}$ and control CD4$^+$ T cells under stimulation of different dosage of TGF-β for 72 hours.

(D-E) (D) Representative flow cytometry analysis and (E) quantification of RORgt and Heilos expression in CD4$^+$Foxp3$^+$ cells in LP of *Ctsw*$^{fl/fl}$ and *Foxp3*^{cre}*Ctsw*$^{fl/fl}$ mice.

Data are representative of at least two independent experiments (B-E). NS, not significant; **p<0.01, ***p<0.001, (B, Two-way ANOVA with Tukey's multiple comparison test; E, Student's *t*-test, error bars represent SD).

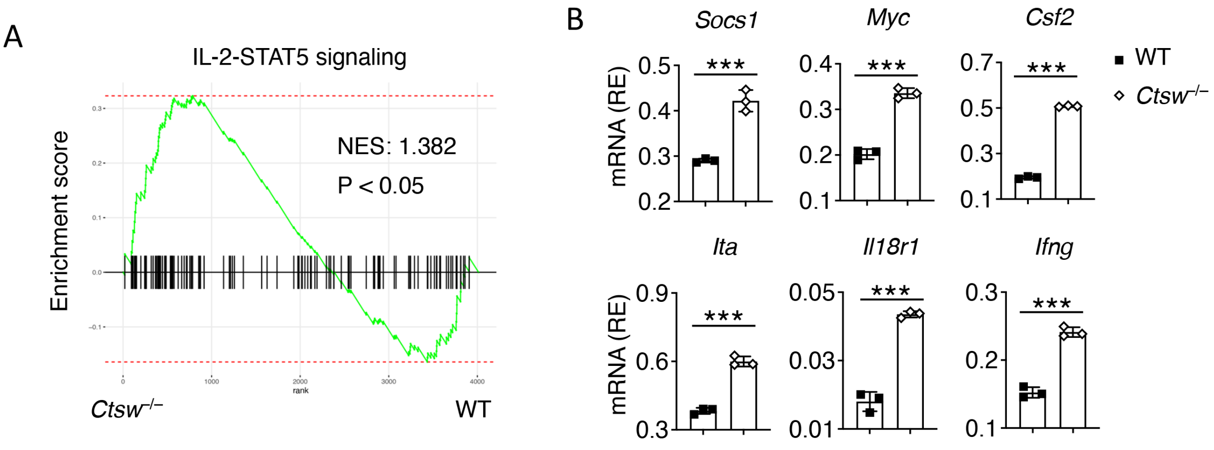

Fig S3. Loss of CTSW leads to compromised IL-2-STAT5 signaling pathway in pTreg cells.

(A) GSEA analysis of IL-2-STAT5 signaling pathway in WT and *Ctsw*$^{-/-}$ pTreg cells.

(B) qPCR analysis of indicated genes from WT and *Ctsw*$^{-/-}$ pTreg cells.

Data are representative of two independent experiments. ***p<0.001, (B, Student's *t*-test; error bars represent SD).

Table S1 Antibodies

**Antibody**	**Source**	**Catalog Number**	**RRID**
**Antibody for Western**			
anti-Flag	Sigma-Aldrich	F1804	AB_262044
anti-V5	Thermo Fisher	R960-25	AB_2556564
anti-Phospho-Stat5 (Tyr694)	Cell Signaling	4322	AB_10544692
anti-STAT5	Cell Signaling	94205	AB_2737403
anti-CD25	BD	553068	AB_394600
anti-β-actin	Sigma-Aldrich	A5441	AB_476744
**Antibody for flow cytometry**			
anti-CTSW	Cloud-Clone	PAC968Mu01	AB_2935896
Alexa Fluor 555 anti-rabbit	Thermo Fisher	A31572	AB_162543
APC/Cy7 anti-CD45	BioLegend	103154	AB_2572116
FITC anti-CD45.1	BD	553775	AB_395043
BV421 anti-CD45.2	BD	562895	AB_2737873
PerCP/Cy5.5 anti-CD4	BioLegend	100540	AB_10372505
PE/Cy7 anti-TCRβ	BioLegend	109222	AB_893625
anti-CD25	Sigma	ZRB1412	AB_2936207
Alexa647 anti-Rabbit IgG	Thermo Fisher	A31573	AB_2536183
PE/Cy7 anti-CD122	Thermo Fisher	25-1222-82	AB_2573390
PE anti-CD132	BD	554457	AB_395404
APC anti-Foxp3	Thermo Fisher	17-5773-82	AB_469457
FITC anti-Foxp3	Thermo Fisher	11-5773-82	AB_465243
PECy7 anti-Helios	BioLegend	137236	AB_2565990
APC anti-Rorγt	Thermo Fisher	17-6988-82	AB_10609207
PE/Cy7 anti-Nrp1	BioLegend	145211	AB_2562359
FITC anti-Thy1.1	Thermo Fisher	11-0900-81	AB_465151
APC anti-hCD2	Thermo Fisher	17-0029-42	AB_10805740
FITC anti-CD44	BioLegend	103006	AB_312957
PE anti-CD62L	BioLegend	104408	AB_313095
PE anti-CD45RB	BioLegend	103308	AB_313015
APC anti-IFNγ	BioLegend	505810	AB_315404
APC anti-IL-4	BioLegend	504106	AB_315320
PE anti-IL-17A	BioLegend	506904	AB_315464
PE anti-IL-13	Thermo Fisher	12-7133-82	AB_763559
**Antibody for immunofluorescence**			
Abberior STAR ORANGE anti-rat	Abberior	STORANGE-1007	AB_2833017
STAR RED anti-mouse	Abberior	STRED-1001	AB_2810982

Table S2 Q-PCR primers

Target gene	Forward	Reverse
Foxp3	CAGCTGGAGCTGGAAAAGGA	CACTGCCCTGAGTACTGGTG
Ctsw	TGTGGGACGCATATCTAACTGT	CTTGATGACACCCTTCTGGTA
Nt5e	GCAGCATTCCTGAAGATGCG	CTCCCGAGTTCCTGGGTAGA
Socs2	TCCAGATGTGCAAGGATAAACG	AGGTACAGGTGAACAGTCCCATT
Il4	GGTCTCAACCCCCAGCTAGT	GCCGATGATCTCTCTCAAGTGAT
Il12rb2	CTCTTTCCATTTTTGCATCAAGTTC	CACCACCGAAGATGAGTGGG
Gzma	CCTGCAATGGGGATTCTGGC	GTATAGACACCAGGCCATCGG
Gamb	GACCCAGCAAGTCATCCCTA	CCAGCCACATAGCACACATC
Socs1	CACCTTCTTGGTGCGCG	AAGCCATCTTCACGCTGAGC
Myc	TTGAAGGCTGGATTTCCTTTGGGC	TCGTCGCAGATGAAATAGGGCTGT
Csf2	ATCAAAGAAGCCCTGAACCTCC	CCCGTAGACCCTGCTCGAATAT
Lta	CTCAGAAGCACTTGACCCAT	TCTCCAGAGCAGTGAGTTCT
Il18r1	ACTTTTGCTGTGGAGACGTTAC	CCGGCTTTTCTCTATCAGTGAAT
Ifng	CTGCTGATGGGAGGAGATGTCT	TGCTGTCTGGCCTGCTGTTA
β-Actin	CAGCTCAGTAACAGTCCGCC	GATCAAGATCATTGCTCCTCCTGA

Table S3 Primers for plasmid cloning

Target	Forward	Reverse
FL Ctsw	TCGGAATTCGCCACCATGACACTGACTG	ATGCTCGAGTCAATGGTGATGGTGATGATGAC
Act Ctsw	GTTAGCGGGCCAAGGCCTCAGTGTGCCCCGCACCTGTGAC	GTCACAGGTGCGGGGCACACTGAGGCCTTGGCCCGCTAAC
Ctsw 151A	GAAGCTGCAAATGCGCCTGGGCCATGGCAGCTGCCGAC	GTCGGCAGCTGCCATGGCCCAGGCGCATTTGCAGCTTC
CD25	ACCCAGTTGTCGGGCAGACTACAAAGACGATGACGACAAGGCAGAACTGTGTCTGTATG	CATACAGACACAGTTCTGCCTTGTCGTCATCGTCTTTGTAGTCTGCCCGACAACTGGGT
CD25RR	TGAATGCAAGAGAGGTTTCGCAGCTCTAAAGGAATTGGTCTATATG	CATATAGACCAATTCCTTTAGAGCTGCGAAACCTCTCTTGCATTCA
